# Structural and biochemical studies of the glucuronoyl esterase *Ot*CE15A illuminate its interaction with lignocellulosic components

**DOI:** 10.1074/jbc.RA119.011435

**Published:** 2019-11-18

**Authors:** Scott Mazurkewich, Jens-Christian N. Poulsen, Leila Lo Leggio, Johan Larsbrink

**Affiliations:** ‡ Wallenberg Wood Science Center, Division of Industrial Biotechnology, Department of Biology and Biological Engineering, Chalmers University of Technology, SE-412 96 Gothenburg, Sweden; § Department of Chemistry, University of Copenhagen, DK-2100 Copenhagen, Denmark

**Keywords:** enzyme structure, enzyme mutation, enzyme kinetics, carbohydrate processing, cell wall, alpha/beta-hydrolase, carbohydrate-active enzymes, catalytic triad, glucuronoyl esterase, lignin-carbohydrate complexes, glucuronoxylan, biomass

## Abstract

Glucuronoyl esterases (GEs) catalyze the cleavage of ester linkages between lignin and glucuronic acid moieties on glucuronoxylan in plant biomass. As such, GEs represent promising biochemical tools in industrial processing of these recalcitrant resources. However, details on how GEs interact and catalyze degradation of their natural substrates are sparse, calling for thorough enzyme structure-function studies. Presented here is a structural and mechanistic investigation of the bacterial GE *Ot*CE15A. GEs belong to the carbohydrate esterase family 15 (CE15), which is in turn part of the larger α/β-hydrolase superfamily. GEs contain a Ser-His-Asp/Glu catalytic triad, but the location of the catalytic acid in GEs has been shown to be variable, and *Ot*CE15A possesses two putative catalytic acidic residues in the active site. Through site-directed mutagenesis, we demonstrate that these residues are functionally redundant, possibly indicating the evolutionary route toward new functionalities within the family. Structures determined with glucuronate, in both native and covalently bound intermediate states, and galacturonate provide insights into the catalytic mechanism of CE15. A structure of *Ot*CE15A with the glucuronoxylooligosaccharide 2^3^-(4-*O*-methyl-α-d-glucuronyl)-xylotriose (commonly referred to as XUX) shows that the enzyme can indeed interact with polysaccharides from the plant cell wall, and an additional structure with the disaccharide xylobiose revealed a surface binding site that could possibly indicate a recognition mechanism for long glucuronoxylan chains. Collectively, the results indicate that *Ot*CE15A, and likely most of the CE15 family, can utilize esters of glucuronoxylooligosaccharides and support the proposal that these enzymes work on lignin-carbohydrate complexes in plant biomass.

## Introduction

Glucuronyl esterases (GEs)^4^ are enzymes able to cleave ester linkages connecting lignin to glucuronoxylan in the plant cell wall. GEs are promising tools for improving biomass-processing technologies by reducing the recalcitrance of so-called lignin-carbohydrate complexes (LCCs) in lignocellulosic biomass (1). Since the characterization of the first fungal GE, *St*GE2 from *Schizophyllum commune* (2), GEs have now been identified in many biomass-degrading microbes including fungi and bacteria and are classified into carbohydrate esterase family 15 (CE15) in the carbohydrate-active enzymes database (CAZy; www.cazy.org (3)). Although great strides have been made in recent years to advance our understanding of GEs, little is known about their molecular interactions with substrates and products and about the nature of the biological substrates they target in the plant cell wall.

GEs belong to the α/β-hydrolase superfamily and possess a catalytic triad common with serine-type hydrolases. All of the CE15 members characterized to date exhibit glucuronoyl esterase activity on various model substrates. Activity on LCCs extracted from woody biomass has also been demonstrated in a few cases (4–6). In recent work, we have revealed that some bacterial CE15 members have more promiscuous substrate specificities, where certain enzymes are able to hydrolyze both glucuronoate and galacturonoate (GalA) esters (7). The first structurally and biochemically characterized CE15 members were of fungal origins, and only recently have bacterial CE15 members been investigated (7–11). The only GE protein structure currently deposited with a ligand is *St*GE2, a variant containing an alanine substitution of the catalytic serine, which is in complex with a methyl ester of the monosaccharide 4-*O*-methyl glucuronate (4-*O*-MeGlcA) (8). As GEs have been shown to act on LCCs, to liberate glucuronoxylan from lignin for further metabolism, the physiological substrate for GEs is most likely glucuronoxylan or glucuronoxylooligosaccharides rather than small 4-*O*-MeGlcA moieties (4–6). Our recent characterization of the bacterial GE *Tt*CE15A from *Teredinibacter turnerae* showed that GEs are indeed able to interact with oligosaccharides (xylotriose appended with 4-*O*-MeGlcA) and that this interaction is mediated by key residues in the active site (11). However, direct molecular evidence of GE-xylan interactions are still lacking, and further structural information is needed.

Consistent with serine-type hydrolases, members of the CE15 family contain a Ser-His-Asp/Glu catalytic triad. However, recent characterizations of bacterial CE15 members have revealed how the location of acidic residue of the catalytic triad is not conserved among GEs. In fungal GEs, the catalytic acid is located in a noncanonical position relative to typical α/β-hydrolases, but in the structures of the bacterial CE15 members MZ0003, discovered in a marine arctic metagenome, and *Tt*CE15A the putative acidic residue was located on the canonical loop (10, 11). In *Tt*CE15A, substitution of its catalytic acid residue to alanine led to a >30-fold reduction in turnover rate, and, interestingly, full activity could not be rescued in the variant by introducing the acidic residue at the noncanonical position exhibited in fungal and many other bacterial GEs. Sequence analysis of CE15 has revealed that several members, such as *Ot*CE15A from the soil bacterium *Opitutus terrae*, have acidic residues at both the canonical and noncanonical positions, raising questions regarding the identity of the catalytic acid in these enzymes (7). Additionally, the observation of CE15 members with acidic residues at both positions may implicate evolutionary transitions between the two positions that could affect interactions with substrates and/or products.

Here, we have investigated by mutagenesis the residues involved in catalysis and carbohydrate binding in the enzyme *Ot*CE15A and, furthermore, present a series of structures of *Ot*CE15A with different ligands, including monosaccharides and, for the first time in CE15, di- and oligosaccharides. We confirmed the importance of a Ser-His-Asp/Glu catalytic triad in the enzyme and showed that both of its putative acidic residues can play a role in substrate hydrolysis. We also determined structures of the enzyme with GlcA, in both native and covalently bound intermediate states, which give insights into the catalytic mechanism of the enzyme and the CE15 family as a whole. Attempts to trap the model substrate benzyl glucuronoate (BnzGlcA) in the active site were unsuccessful, although a structure was determined with BnzGlcA found in a site on the edge of the active site cleft that may indicate the route of the substrate prior to hydrolysis. We also determined a structure of *Ot*CE15A in complex with GalA, and we show how this promiscuous activity is made possible in the enzyme. Finally, structures determined with the tetrasaccharide 2^3^-(4-*O*-methyl-α-d-glucuronyl)-xylotriose and the disaccharide xylobiose showcase how the enzyme can indeed act on glucuronoxylan-derived fragments. Collectively, the results advance our understanding of the CE15 family as a whole and provide useful insights for further developing biochemical biomass conversion technologies.

## Results

### Kinetic characterization of catalytic residue substitutions

**Figure 1.**
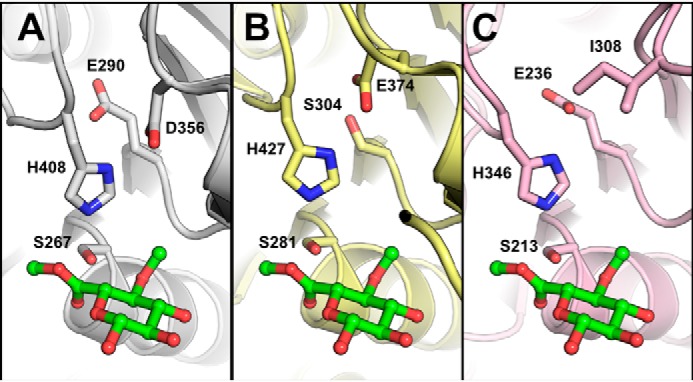
**Catalytic residues of *Ot*CE15A and other CE15 members.** Comparison of the catalytic triads of *Ot*CE15A (PDB code 6GS0; *A*), *Tt*CE15A from *T. turnerae* (PDB code 6HSW; *B*), and *St*GE2 from *Thermothelomyces thermophila* (previously *Sporotrichum thermophile*; PDB code 4G4G; *C*) shows alternate positions of the catalytic acid residue in *Tt*CE15A and *St*GE2, whereas an acidic residue is present in both locations in *Ot*CE15A. The methyl ester of 4-*O*-methyl glucuronoate determined in the structure of *St*GE2 (PDB code 4G4J) is shown in *green sticks* from structural alignment of the CE15 proteins.

The activity of *Ot*CE15A and nine other bacterial CE15 members has previously been characterized on model substrates, laying the foundation for these mechanistic studies (7) (Table S1). All CE15 members contain a catalytic triad comprised of Ser-His-Glu/Asp, as found in other serine-type hydrolases. Substitution of the catalytic residues in many serine-type hydrolases has resulted in significantly compromised but detectable activity in several enzyme variants (12, 13). Here, we have quantitatively assessed the effects following substitution of all catalytic residues of the CE15 enzyme *Ot*CE15A using BnzGlcA as a model substrate (Table 1). Substitution of either the catalytic serine (Ser-267) or histidine (His-408) with alanine significantly compromised the *Ot*CE15A turnover rate (*k*_cat_), reducing the rate by 17,000- and 1,700-fold, respectively. Compared with the *Tt*CE15A S281A variant, where the *k*_cat_ only decreased 370-fold (11), the S267A variant of *Ot*CE15A was dramatically crippled. *Ot*CE15A is an interesting member of CE15 and the α/β-hydrolase superfamily in that it features acidic residues at both the canonical and noncanonical positions (Glu-290 and Asp-356) (Fig. 1). Previous investigations of *Tt*CE15A showed how attempting to shift its single catalytic acid from the canonical to the noncanonical position resulted in a severely crippled enzyme regarding both *k*_cat_ and *K_m_* (11). Intriguingly, substitution of either acidic residue in *Ot*CE15A with alanine resulted in only a 1.5- or 3-fold decrease in *k*_cat_ (Table 1), and only upon substitution of both acidic residues was the *k*_cat_ decreased by a similar degree as that of the E374A variant of *Tt*CE15A (100-fold *versus* 33-fold, respectively). The results collectively imply that whereas one of the residues may be the principle acidic residue in the catalytic mechanism, the other residue is functionally redundant.

**Table 1:** **Kinetic parameters of BnzGlcA esterase activity of the WT *Ot*CE15A and alanine variants of its catalytic residues** Errors represent S.D. from triplicate experiments

*Ot*CE15A	*K_m_*	*k*_cat_	*k*_cat_*/K_m_*
	*mm*	*s*^−*1*^	*s*^−*1*^ *mm*^−*1*^
WT	3.57 ± 0.091	16.6 ± 0.11	(4.65 ± 0.12) × 10^3^
S267A	2.83 ± 0.66	0.000983 ± 0.000059	(3.47 ± 0.84) × 10^−1^
H408A	0.184 ± 0.035	0.00999 ± 0.00040	(5.43 ± 1.06) × 10^1^
E290A	2.03 ± 0.11	10.3 ± 0.11	(5.07 ± 0.28) × 10^3^
D356A	1.86 ± 0.11	5.21 ± 0.073	(2.80 ± 0.17) × 10^3^
E290A/D356A	0.502 ± 0.034	0.196 ± 0.0025	(3.90 ± 0.27) × 10^2^

### Catalytic OtCE15A variants in complex with GlcA and benzyl glucuronoate

**Figure 2.**
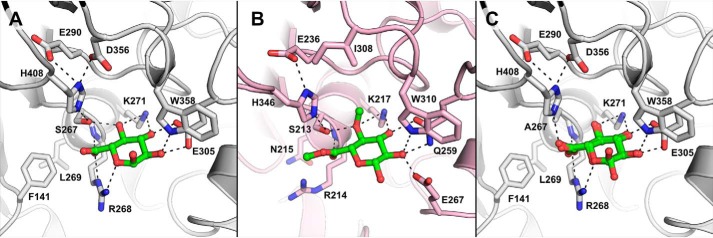
**Glucuronate bound to *Ot*CE15A.** Binding of glucuronate to the WT *Ot*CE15A (*A*; PDB code 6SYR) and S267A *Ot*CE15A variant (*C*; showing both anomers of the carbohydrate; PDB code 6SYV) is similar to that of the methyl ester of 4-*O*-methyl glucuoronoate determined in the structure of *St*GE2 from *T. thermophila* (*B*; previously *S. thermophile*; PDB code 4G4G with the ligand taken from 4G4J by structural alignment). Notably, the side chain of the conserved active-site arginine is found rotated toward, and interacts with, the glucuronate molecule in the *Ot*CE15A product ligated structures differently than in the *St*GE2 substrate ligated structure.

To advance the understanding of the interactions between enzyme, substrates, and products in CE15, we pursued structures of *Ot*CE15A, and its variants, in complex with various ligands. We determined the structure of the WT *Ot*CE15A in complex with the product GlcA (Fig. 2*A*). Relative to the apo-structure previously determined, binding of GlcA resulted in minimal changes in the enzyme's structure (all atom root mean square deviation of 0.639 Å), and the binding of the uronic acid to *Ot*CE15A is very similar to that seen in the structure of the fungal *St*GE2 (catalytic S213A variant) in complex with the methyl ester of 4-*O*-MeGlcA (Fig. 2*B*) (8). In *Ot*CE15A, the GlcA is positioned by a series of hydrogen bonds: one between the C2 hydroxyl and Trp-358, two between the C2 and C3 hydroxyls with Glu-305, two between the C3 and C4 hydroxyls with Lys-271, and one between C4 hydroxyl and the catalytic serine (Ser-267). The C5 carboxylate is oriented similarly to that observed in the *St*GE2 complex structure and makes a hydrogen bond to the main-chain nitrogen of a conserved active-site arginine (Arg-268 in *Ot*CE15A and Arg-214 in *St*GE2), which, as for most α/β-hydrolases, is likely to contribute to forming the oxyanion hole to stabilize the negatively charged transition state during catalysis. A notable difference from the *St*GE2 complex structure, however, is that the side chain of the conserved arginine in *Ot*CE15A is oriented toward the glucuronic acid and makes two additional hydrogens bonds: one between O5 of the pyranose ring and one of the arginine's Nη atoms and one between the carboxylate and the arginine's Nϵ. Thus, this side chain almost certainly contributes to formation of the oxyanion hole. In all structurally characterized bacterial GEs, the arginine appears to be locked in place by a conserved aspartate (Asp-207 in *Ot*CE15A), whereas the equivalent residue in the two solved fungal structures is a glutamine and results in the arginine side chain in those structures being rotated away from the uronic acid–binding site.

**Figure 3.**
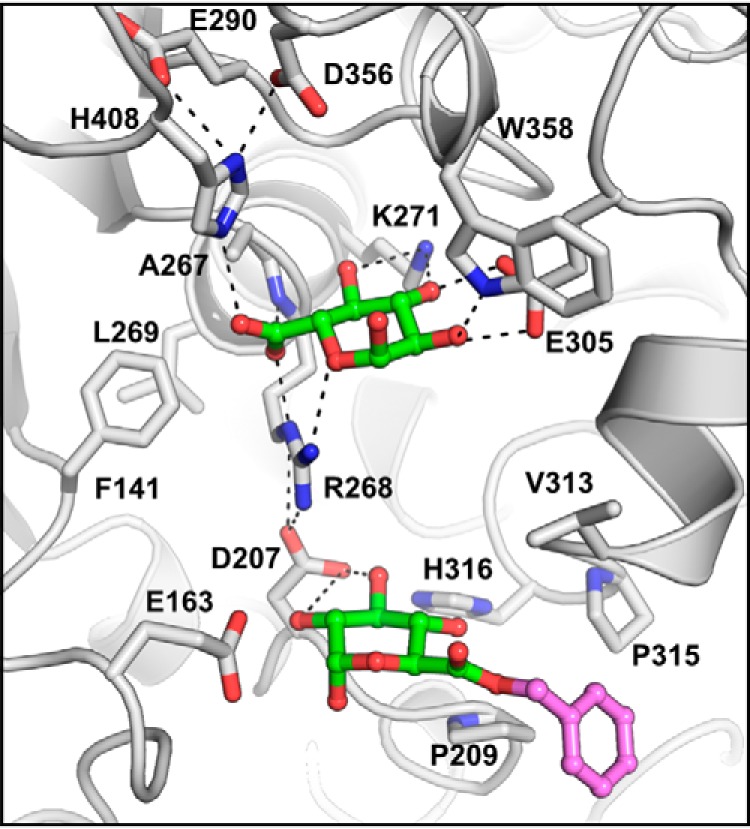
**Benzyl glucuronoate trapped on the surface of the S267A *Ot*CE15A variant (PDB code 6T0E).** Presumably, the cleavage rate was too fast to capture the Michaelis–Menten complex with benzyl glucuronoate over the short crystal soaking period (10 s) but allowed capture of the uncleaved substrate along the edge of the active site cleft ∼10 Å from the catalytic center (glucuronate portion in *green* and benzyl portion in *magenta*).

To investigate the substrate binding in *Ot*CE15A, we sought to obtain complex structures of the catalytically impaired S267A enzyme variant with GlcA and the model substrate BnzGlcA, respectively. An S267A-GlcA complex structure was determined to high resolution (1.12 Å) and showed that in the absence of the catalytic serine, the glucuronate binds identically as in the WT protein (Fig. 2*C*). Notably, this high-resolution structure facilitated the observation of both glucuronate anomers being present. Despite several attempts, we were unable to trap the Michaelis complex with BnzGlcA in our experiments as, presumably, the activity of the S267A variant was still too high to preserve the intact substrate in the active site even in short soaks (as short as ∼5 s). However, we were able to obtain a structure with GlcA bound in both active sites (for this particular complex, a crystal form with two molecules in the asymmetric units was used) and an additional BnzGlcA molecule near one of the active sites (Fig. 3). The substrate molecule is located along the edge of the active site cleft, making only one hydrogen bond between its C3 hydroxyl and the carboxylate of Asp-207. Although making minimal interactions with the protein, its close proximity to the active site makes this interaction interesting, as it could indicate a mechanism for substrate recognition and a route into the active-site cleft.

**Figure 4.**
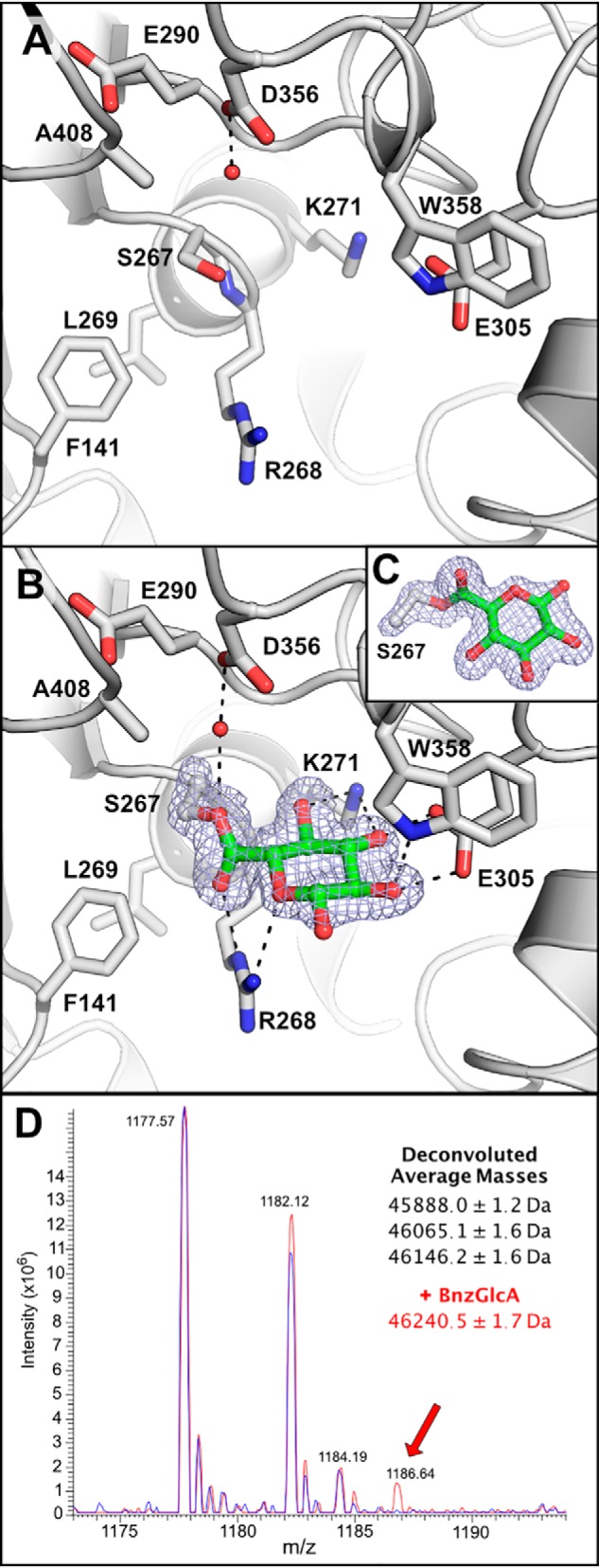
**Trapping the glucuronate covalent intermediate in the *Ot*CE15A H408A variant.** Shown is the *Ot*CE15A H408A variant in the absence (*A*; PDB code 6SZ0) and presence of benzyl glucuronoate (*B*; PDB code 6SZ4). Presumably, the acylation rate was too fast to capture the Michaelis–Menten complex with benzyl glucuronoate over the short crystal soaking period (5 s) but allowed capture of the acyl-enzyme intermediate with the glucuronate moiety covalently linked to the catalytic nucleophile Ser-267. In both structures, a water molecule, hydrogen-bonded to Asp-356, fills the void left from substitution of the catalytic histidine. The covalent serine-glucuronoyl adduct is shown with the density from an omit map, at 4σ, created in Phenix (39) by omitting GlcA and the Cα, Cβ, and Oγ of Ser-267. *C*, an alternate orientation of the serine-glucuronoyl adduct showing the linkage. *D*, mass spectrum of the *Ot*CE15A H408A variant in the absence (*blue*) and presence of benzyl glucuronate (*red*) leads to the production of a new mass consistent with the glucuronate covalent intermediate.

We also pursued a complex structure of a catalytic histidine mutant (H408A) with BnzGlcA. We determined the structure of the H408A variant in the absence of substrate and saw only minimal changes in the protein structure (Fig. 4*A*). As for the S267A variant, despite several attempts, we were unable to trap the Michaelis complex with BnzGlcA in the H408A variant. However, following a 5-s substrate soak, we were able to determine the structure of the product GlcA covalently bound to the catalytic serine (Ser-267) through the C5 carboxylate (Fig. 4*B*). All active-site residues in this complex structure, including the catalytic serine, are in equivalent positions as in the WT structures. However, the GlcA is slightly rotated along an axis between the C2 and C3, leading to the C6 moving 1.2 Å closer to the catalytic serine and enabling the linkage to the serine oxygen. The other C6 oxygen, now the carbonyl of the acyl intermediate, is still positioned by the conserved active site arginine and maintains its interactions with the residue through both its main-chain nitrogen and Nϵ atoms. To complete the esterase reaction, a water molecule, presumably from the bulk solvent, would come in and attack the linkage at the C6 position.

This is the first structure of a trapped covalent intermediate in a CE15 member. The result was quite unexpected, as without the histidine, the catalytic serine is expected to be a poor nucleophile. In the WT protein, decomposition of the covalent intermediate is assumed to be quick because the substrate turnover rate is high and only noncovalently linked GlcA products have been observed in previously determined structures. To explore whether the covalent intermediate could be detected in solution with the H408A variant and was not an artifact of crystallization, we determined the mass of the protein using intact-protein MS, in the presence and absence of added BnzGlcA. In the absence of added substrate, the protein sample resulted in three prominent masses (45,888.0 ± 1.2, 46,065.1 ± 1.6, and 46,146.2 ± 1.6 Da), with the lowest mass closely resembling the expected mass of 45,889.38 of the protein lacking the N-terminal methionine (Fig. 4*C*). The heavier masses differ from the smallest by +178 and +258 Da and are consistent with acylation of the protein with gluconoyl and 6-phosphogluconoyl groups, respectively, similar to those previously observed with other His-tagged proteins overexpressed in *Escherichia coli* (14). In the presence of BnzGlcA, a new signal of 46,240.5 ± 1.7 Da appears and is consistent with the glucuronidation of the gluconoyl-modified protein. Note that the mass change due to glucuronidation (+176) is too similar to the mass of the gluconoyl modification (+178) and prevents the detection of glucuronidation of the unmodified protein directly. Although the MS methodology is not quantitative, qualitatively we can conclude that not all enzyme molecules are glucuronidated, which is not highly surprising because the H408A enzyme variant retains some activity and thus detection of only a proportion of the covalent intermediate would be expected. Taken together, the detection of the covalent intermediate in solution indicates that the intermediate in the crystal structure is not an artifact and likely indicates that the rate of deacylation is significantly decreased in this enzyme variant, which enables its detection.

### OtCE15A in complex with GalA

**Figure 5.**
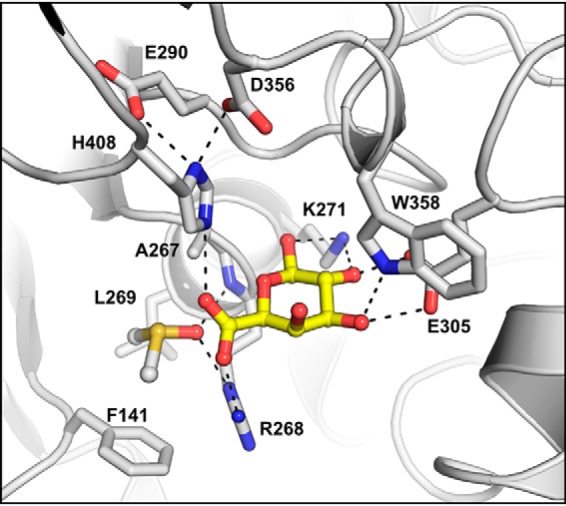
**Galacturonate bound to the *Ot*CE15A S267A variant (PDB code 6SZO).** Presumably, the cleavage rate was too fast to capture a Michaelis–Menten complex with methyl galacturonoate over the crystal soaking period (60 s) but allowed capture of the galacturonate product in the active site. The galacturonate is shown in *yellow sticks*, and a DMSO molecule, used as a solvent for the substrate, present in the oxyanion hole is shown in *sticks*.

Previous characterizations of *Ot*CE15A and other bacterial CE15 members revealed that some enzymes are able to use MeGalA as a substrate with specificity constants similar to those for MeGlcA (7). To elucidate how *Ot*CE15A could facilitate this reaction, we soaked crystals of the S267A variant with MeGalA. Although we were unsuccessful in obtaining the Michaelis complex, we were able to determine a 1.34 Å structure of the *Ot*CE15A in complex with the product of the reaction, GalA, and the configuration was quite distinct compared with that of GlcA. The distinction principally lies in the pyranose being flipped relative to the C6, which results in the anomeric hydroxyl group being bound in the same position as the C4 of GlcA in previous structures (Fig. 5). The β anomer of GalA has been modeled here with its hydroxyl interacting with Lys-271. Whereas the flipped pyranose results in the position of the C2 and C3 being swapped, the equatorial hydroxyl groups from both carbons are positioned similarly as in the nonflipped GlcA structure. The C4 hydroxyl group projects out of the cleft and possibly makes a weak/long-distance hydrogen bond with Trp-358 (3.5 Å). Last, the C6 carboxylate is positioned close to where the GlcA carboxylate is found in other structures, but in a different orientation, as one of the oxygen atoms forms hydrogen bonds with the Nϵ2 of His-408 and the other with one of Arg-268's Nη atoms.

The interaction between one of the GlcA carboxylate oxygens and Arg-268, through the Nϵ and main-chain amino group, is absent in the GalA structure, and instead hydrogen bonds are formed between these atoms and the carbonyl of a DMSO solvent molecule. Although it is possible that the positioning of the GalA C6 carboxylate observed here is an artifact of the S267A substitution, the S267A structure determined with GlcA still shows the C6 carboxylate positioned in the same way as in the WT enzyme. Thus, it is likely that the observed configuration is biologically relevant and facilitates catalysis. It is noteworthy that the residue Phe-141, previously proposed to interact with lignin-derived aromatics in bacterial CE15 members (7), is rotated away from the active site to accommodate the binding of DMSO and exposes a leucine (Leu-269) in the pocket. Interestingly, sequence analysis of CE15 members revealed a correlation between MeGalA activity and the presence of a Leu or small residue at the equivalent position of Leu-269 in *Ot*CE15A (Fig. S1). CE15 members such as *Ot*CE15D and CE15B from *Solibacter usitatus* (*Su*CE15B), which lack MeGalA activity, have a large residue at the equivalent position and could contribute to defining the substrate specificity. To explore this hypothesis, we determined the effects of substitution of the *Ot*CE15A Leu-269 with tyrosine on the enzyme's MeGlcA and MeGalA activities (Table 2). Introduction of a tyrosine at this position did indeed perturb the enzyme's activity with MeGalA, increasing the *K_m_* 10-fold and decreasing the *k*_cat_ 6-fold relative to its MeGlcA activity. However, the effects were not as pronounced as expected relative to the huge differences in MeGlcA *versus* MeGalA activity observed in CE15 homologs containing a similar residue at this position. Thus, these results suggest that the type of residue at this position contributes to MeGalA specificity in *Ot*CE15A. However, the less pronounced effects of the residue substitution suggest that either other undefined determinants contribute to the lack of MeGalA activity in other CE15 members or introduction of a tyrosine at the Leu-269 position in *Ot*CE15A is insufficient to replicate the same architecture found in other CE15 homologs.

**Table 2:** **The effect of the L269Y subsitution on the kinetic parameters of *Ot*CE15A with model substrates** Esterase activity with methyl esters of GlcA and GalA are shown. Errors represent S.D. from triplicate experiments. *K_si_*, substrate inhibition constant. NA, not applicable.

Enzyme	Substrate	*K_m_*	*K_si_*	*k*_cat_	*k*_cat_*/K_m_*
		*mm*	*mm*	*s*^−*1*^	*s*^−*1*^ *m*^−*1*^
WT	MeGlcA	2.77 ± 0.15	NA	19.0 ± 0.31	(6.85 ± 0.39) × 10^3^
	MeGalA	5.31 ± 0.51	NA	28.8 ± 0.64	(4.85 ± 0.47) × 10^3^
L269Y	MeGlcA	1.69 ± 0.48	34.5 ± 11.9	16.1 ± 2.1	(9.54 ± 3.0) × 10^3^
	MeGalA	11.9 ± 0.60	NA	2.70 ± 0.052	(2.26 ± 0.12) × 10^2^

### OtCE15A in complex with oligosaccharides

Although the biological substrates of CE15 enzymes are believed to be glucuronoxylan ester–linked to lignin, how the enzymes interact with this substrate remains elusive. To investigate the interaction between *Ot*CE15A and glucuronoxylan, we performed soaking experiments with various glucuronoxylan-derived oligosaccharides produced from partial enzymatic digestion of xylan. Following several soaking experiments, we were able to determine structures of *Ot*CE15A with the tetra-saccharide 2^3^-(4-*O*-methyl-α-d-glucuronyl)-xylotriose (commonly referred to as XUX), which is the largest ligand in a CE15 protein structure to date.

**Figure 6.**
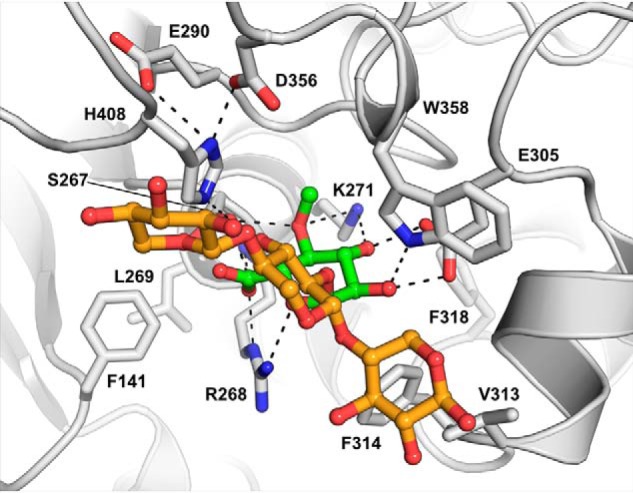
**OtCE15A in complex with the glucuronoxylan oligosaccharide XUX (PDB code 6T0I).** The oligosaccharide, produced from beech xylan as described under “Experimental procedures,” is shown with the 4-*O*-methyl-α-d-glucuronate moiety in *green sticks* and the xylotriose moiety in *orange sticks*.

The 4-*O*-MeGlcA moiety of XUX binds in a very similar way as the single GlcA in the WT and S267A *Ot*CE15A structures (Fig. 6), although with loss of a water molecule, normally hydrogen-bonding with the C4 hydroxyl. The binding of the 4-*O*-MeGlcA moiety is highly similar to that seen in the *St*GE2 structure complexed with 4-*O*-methyl-d-glucuronoate. Together, the observations indicate that *Ot*CE15A does not discriminate between glucuronoxylans containing 4-*O*-methylated or nonmethylated GlcA residues in glucuronoxylan. This is a significant finding, as previous reports of fungal GEs have indicated that those enzymes require the 4-*O*-methyl substitution for activity (15–18), and thus this difference in substrate specificity could be a significant distinction across the CE15 family. However, more extensive comparative analyses between bacterial and fungal CE15 members are required to fully elucidate this difference in specificity.

In the XUX complex structure, besides interactions with the 4-*O*-MeGlcA, there is a lack of polar interactions between the protein and the oligosaccharide. Instead, the xylotriose portion of the oligosaccharide packs across the upper face of the cleft, burying 579 Å^2^ of solvent-accessible surface area and bridging across residues Val-313, Phe-314, Trp-358, and His-408. Previous docking simulations with *Ot*CE15A and inhibition studies of *Tt*CE15A with XUX have implicated the conserved active-site tryptophan (Trp-358 in *Ot*CE15A) as a major contributor to the interactions with xylooligosaccharides (7, 11). Here, we can directly validate that Trp-358 contributes to binding of glucuronoxylooligosaccharides. A symmetry-related protein molecule is closely positioned to the active site, and the xylose moiety on the reducing end is found to interact with two residues of this symmetry-related protein molecule: Nϵ2 of His-56 with the O1 and O2 and the carbonyl of Gln-55 with the O2. The position of the symmetry mate would sterically restrict binding of longer gluronoxylooligosaccharides extended from the reducing end and may be slightly distorting the orientation of the xylan chain of XUX observed here. However, the close packing of the xylose units and the evidence from previous biochemical studies suggest that the binding position is biologically relevant. Future structural studies, potentially of other CE15 enzymes in complex with XUX or other glucuronoxylan-derived oligosaccharides, would be highly informative to possibly corroborate this result.

### OtCE15A in complex with xylobiose

**Figure 7.**
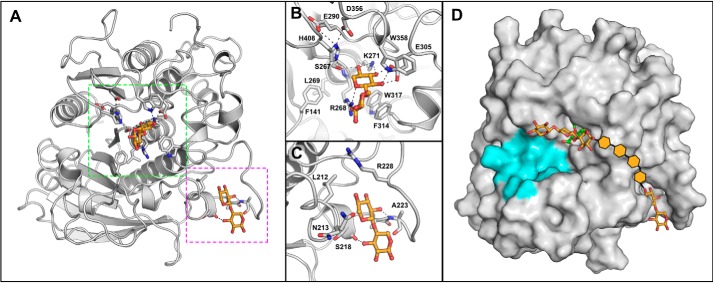
**OtCE15A in complex with xylobiose.** Shown is the overall structure of *Ot*CE15A, showing the two xylobiose-binding sites (PDB code 6SYU) highlighted in *green* and *magenta* (*A*) for the active site (*B*) and secondary site (*C*), respectively. The secondary site is ∼25 Å from the active site. *D*, a proposal of how the secondary xylobiose site could connect to the XUX found in the active site. Potential xylose moieties connecting the xylobiose observed in the second site to the XUX molecule observed in the active site are shown by orange hexagons. The region of the protein proposed to interact with lignin is colored in cyan.

To gain further insight into substrate binding, we pursued soaking of corn cob xylan into *Ot*CE15A. However, analyses of the corn cob xylan used (Sigma) revealed that it lacked any uronic acids (GlcA or 4-*O*-MeGlcA) and that instead of consisting of polysaccharides, it was comprised essentially of short xylooligosaccharide fragments dominated by xylobiose (data not shown). In light of this, the *Ot*CE15A structure determined with two xylobiose molecules upon soaking with the mixture was not surprising, although it serendipitously reveals interesting features of *Ot*CE15A (Fig. 7*A*).

One of the xylobiose molecules is found in the active site of the enzyme with a xylose moiety binding analogously to GlcA and XUX, except for the lack of the C5 carboxylate and the xylobiose being β-linked instead of the α-linked GlcA in XUX (Fig. 7*B*). The presence of xylobiose in the active site suggests that xylose or xylooligosaccharides might inhibit the enzyme reaction. Indeed, kinetic characterization revealed that xylose inhibits the BnzGlcA esterase reaction with a *K_i_* of 48.1 mm (Fig. S2). Analogously, the presence of either 100 mm xylose or xylobiose reduced the enzymatic activity by ∼50%, suggesting a common mechanism of inhibition between the two molecules. Glucose, in concentrations up to 250 mm, does not inhibit the BnzGlcA esterase reaction (<10% inhibition), indicating a specificity for xylooligosaccharides. The inhibition by xylose is likely not biologically relevant however, as xylose concentrations would be unlikely to reach such levels during active cell wall degradation by microorganisms. However, the inhibition could have significant effects industrially, where large scale saccharification of biomass leads to high concentrations of simple sugars that could hamper the esterase reaction.

The second xylobiose is bound in a site located about 25 Å from the active site with the sugar packed between a short helical region and a loop comprised of residues 222–228 (Fig. 7*C*). The xylobiose is modeled such that it packs with the nonreducing end projecting into the cleft and the reducing end projecting away from the protein. The sugar makes only a few interactions with the protein, with only hydrogen bonds between the main-chain amino of Ala-223 and O2 of the nonreducing xylose residue and between the main-chain carbonyl of Ser-218 and O3 of reducing xylose residue. This loop region in other *Ot*CE15A structures (residues 220–227) has large *B*-factors likely resulting from considerable movement of the loop. However, in the presence of xylobiose, the B-factors of this region are reduced significantly. The biological relevance of this binding is unclear, and its presence may be just an artifact of the soaking condition. However, an alternative hypothesis could be that the protein can interact with xylan chains not only in the active site but possibly also in other surface-binding sites similar to those found in other CAZymes (19). However, further investigations are needed to explore these possibilities.

## Discussion

The Michaelis complex with the BnzGlcA proved unobtainable by the methods tested here. A previous study of the CE15 enzyme MZ0003 reported similar difficulties in obtaining the equivalent complex (10). From the ligand complex structures determined here, it has become evident that the conserved arginine in the CE15 family likely makes significant contributions to catalysis by not only positioning the substrate carboxyl group, but also potentially aiding catalysis by stabilizing the oxyanion intermediate (*i.e.* forming the oxyanion hole). This is most evident in the *Ot*CE15A-H408A complex structure with the GlcA covalent intermediate, where the arginine still contributes to the positioning of the carbonyl group. Similar to other α/β-hydrolases, the CE15 members also share some similarity around the stabilization of the oxyanion, aided by the main-chain amide nitrogen at the end of the helical dipole that is proximal to the catalytic serine. However, an arginine residue is rarely found following the catalytic serine in other α/β-hydrolases, not even within the CE15 family's closest relatives, CE7 (comprising acetyl xylan esterases and cephalosporin-C deacetylases). Instead, a variety of other strategies are used across the α/β-hydrolase superfamily to stabilize the oxyanion intermediate. Most commonly utilized, and what is found in CE7, is a loop region with its main-chain amide nitrogen groups projecting toward the catalytic serine, which is positioned analogously to the CE15 conserved arginine side chain (20–23). We suggest, therefore, that the Arg side chain, although slightly differently positioned in the fungal and bacterial structures studied to date, contributes to the transition state stabilization in the catalytic mechanism, explaining its conservation in >95% (26 of 27) of characterized CE15 sequences. In the bacterial CE15 enzymes, the conserved arginine is kept in this position by interaction with an acidic residue not conserved among fungal CE15 members. In both *Ot*CE15A and MZ0003, having the arginine in the active state likely aids in driving the forward cleavage reaction too efficiently to allow trapping of the Michalis complex even in the absence of the catalytic serine. In future endeavors, it would be highly interesting to explore not only the importance of this residue in GE-catalyzed reactions but also substitutions that could possibly enable attainment of Michaelis complexes.

The minimal effect of single substitutions of either potential acidic residue of the *Ot*CE15A catalytic triad showed that these residues are functionally redundant. In CE15, most members carry either one of the acidic residues, and, given the nature of the protein fold and similarities within the family, it is unlikely that this is a case of convergent evolution. Instead, the presence of two functional acidic residues may indicate an evolutionary route toward new functionalities. While the acidic residue of the first characterized α/β-hydrolase superfamily members was found to be at the position equivalent to the *Ot*CE15A Glu-290 (20), more recent investigations of α/β-hydrolase superfamily members have additionally observed the acidic residues at the noncanonical position equivalent to the *Ot*CE15A Asp-356 (24–26). In *Tt*CE15A, which possesses a single catalytic acid, a switch between the two positions did not yield an enzyme with WT activity, and its active site may have adopted a clear specialized configuration (11). Thus, the double acidic residues of *Ot*CE15A may, in contrast, be a state midway between the two observed putatively specialized locations and CE15 enzyme functionalities.

As for other serine α/β-hydrolases, the reaction mechanism of glucuronoyl esterases proceeds through an acyl-enzyme intermediate. The rate-limiting step of the two-step mechanism varies among serine α/β-hydrolases and can be affected by the substrate or biochemical conditions utilized (27). For *Ot*CE15A and other bacterial CE15 enzymes characterized to date (7), a relatively consistent *k*_cat_ when utilizing different esters of GlcA would suggest that it is the deacylation step that is rate-determining in these enzymes. The absence of an acyl-enzyme intermediate in crystallographic studies with the WT *Ot*CE15A indicates that the deacylation rate is too rapid to observe the intermediate on the time scales utilized. However, upon substitution of the catalytic histidine, the detection of the acyl-enzyme by MS and crystallography in addition to the decrease in *K_m_* for GlcA substrates fits well with the accumulation of the covalent intermediate if breakdown of the intermediate is rate-limiting, as has been analogously observed with glycosidase variants (28, 29). Presumably then, the active site architecture, with the oxyanion hole priming substrate for nucleophilic attack, is elegantly constructed for rapid scission of the ester linkage, even when members of the catalytic triad are absent.

The GlcA moieties of glucuronoxylan are not always methylated, and the ratio of their etherification likely varies, depending on a host of factors, such as species, tissues, cell type, etc. (30–33). Whereas several reports have suggested that the 4-*O*-methyl substitution is crucial for activity among fungal CE15 enzymes (15–18), its requirement for activity has not been observed among the bacterial members characterized to date (7, 11). Structures of the *Ot*CE15A enzyme with GlcA and 4-*O*-MeGlcA, as a part of XUX, determined here reveal that the position and interactions with O4 of the bound sugar are the same as those observed in the *St*GE2 (8). However, in the GlcA complex structures, a water molecule is commonly found filling the void left by the absence of methyl substituent and makes a hydrogen bond to the C4 hydroxyl. In fact, there is more space in this corner of the cleft to accommodate potentially a larger substitution at the C4 position, such as an acetylation. However, to the best of our knowledge, no such substitutions occur in nature. Taken together, the results indicate that the *Ot*CE15A (and likely many other CE15 enzymes) is versatile and can utilize both methylated and nonmethylated GlcA substrates in addition to substrates with or without extended carbohydrate portions such as found in glucuronoxylan in plant biomass. *Ot*CE15A can also utilize GalA esters as substrates, unique to a subset of CE15 members, and we showed that it accomplishes this by binding the sugar in a flipped orientation, which to the best of our knowledge is the first time a similar occurrence with carbohydrate active enzymes has been observed.

In this work, we have determined several ligand complex structures with *Ot*CE15A to shed light on the catalytic mechanism and how the CE15 enzymes interact with the carbohydrate portions of their complex substrates. Our structure of *Ot*CE15A with XUX is the first structure of a CE15 protein with a carbohydrate larger than a monosaccharide. The structures determined with BnzGlcA, found in a site close to the active site, and xylobiose, with the sugar found in a secondary site ∼25 Å from the active site, could indicate sites on the enzyme for interactions with longer glucuronoxylooligosaccharides (Fig. 7*D*). It remains elusive whether, and how, these enzymes may interact with the lignin portion of their physiological substrates in the plant cell wall, which may be illuminated by further investigations.

## Experimental procedures

### Enzyme production and assays

Enzyme variants were created by site-specific mutagenesis by the QuikChange method using the primers listed in Table S2 (34). *Ot*CE15A enzymes were recombinantly produced in *E. coli* BL21 (λDE3) and purified as described previously (7). Glucuronoyl esterase activity was assayed with BnzGlcA, MeGlcA, or MeGalA (Carbosynth) and monitored continuously with the K-URONIC kit (Megazyme) as reported previously (7). The xylose competitive inhibition constant was determined with BnzGlcA as the substrate with increasing xylose concentrations. Inhibition assays with xylose, xylobiose, and glucose were carried out with BnzGlcA as the substrate at concentrations of 3.5 mm (at the enzyme's *K_m_*) and at 350 μm (10-fold less than the *K_m_*). All kinetic data were fitted using GraphPad Prism 8.

### Production of glucuronoxylan-derived oligosaccharides

The aldouronic acid XUX was produced using a GH30 xylanase from *Bacteroides ovatus* (*Bo*GH30, locus tag BACOVA_03432), which was obtained by cloning, expressing, and purifying the protein as described previously (35). The gene was amplified by PCR using the primers listed in Table S2 and subsequently cloned into a modified pET-28a vector containing a cleavage site for tobacco etch virus protease in place of the native thrombin cleavage site (generously provided by N. Koropatkin, University of Michigan). The protein was produced by overexpression in *E. coli* BL21 (λDE3) and purified by Ni^2+^ immobilized metal affinity chromatography by standard procedures on an ÄKTA Explorer (GE Health Sciences). XUX was produced by *Bo*GH30 digestion of beech xylan (Sigma-Aldrich). Overnight, room temperature digestion reactions (500 μl) were carried out in 25 mm sodium phosphate at pH 7 with 10 mg/ml beech xylan and 0.5 mg/ml purified *Bo*GH30. The enzyme was removed by ultrafiltration through a 10-kDa Amicon spin filter, and the filtrate was dried by lyophilization.

### Crystallization and ligand soaking

Crystals of *Ot*CE15A were prepared by sitting drop (0.3 μl drops) in MRC two-drop crystallization plates (Molecular Dimensions) with protein (at concentrations between 20 and 30 mg/ml) mixed with reservoir solutions in either a 3:1 or 1:1 ratio by aid of an Oryx 8 robot (Douglas Instruments). Crystals utilized for soaking were obtained directly from, or through slight optimization of, crystallization conditions from the JCSG+ or Morpheus crystal screens (Molecular Dimensions) (36, 37), most close to the originally identified crystallization conditions (7). The final crystallization conditions for each data set obtained are summarized in Table S3.

Solutions utilized for soaking experiments with BnzGlcA or MeGalA (Carbosynth) were made by mixing the mother liquor with a saturated solution of substrate, prepared in DMSO. Crystals were then extracted and soaked in 1 μl of the soaking solution for varying time periods before being flash-frozen in liquid nitrogen. The resulting data sets published here had the following soaking conditions: *Ot*CE15A-S267A-BnzGlcA complex was achieved by soaking for 10 s, *Ot*CE15A-S267A-GalA complex was achieved by soaking for 60 s, and the *Ot*CE15A-H408A-GlcA complex was achieved by soaking for 5 s. Soaking of crystals with GlcA, XUX prepared from *Bo*GH30 digestion of beech xylan, and xylobiose from corn cob xylan (Carbosynth) utilized saturated solutions of the compounds raised up in the mother liquor. Crystals were allowed to soak for at least 1 h before being flash-frozen in liquid nitrogen.

### Structure determination and refinement

All of the data sets were processed with XDS (38), and the structures were determined in Phenix (39) by either rigid body refinement with Phenix Refine (40) when isomorphous with the published structure (PDB code 6GS0 (7)) or molecular replacement with Phaser using the same as search model in the case of structures *Ot*CE15A-S267A-GalA (PDB code 6SZO) and *Ot*CE15A-S267A-BnzGlcA (PDB code 6T0E) (41). Coot (42) and Phenix Refine were used in iterative cycles of manual and computational refinement. Where possible, the ligand restraint files were obtained from the CCP4 library (43). The BnzGlcA compound was created in PyMOL, and its restraints were created with Phenix eLBOW (44). The data collection, processing, and refinement statistics for all of the data sets can be found in Table S4. For the 6SYV and 6SYU data sets, although the overall completeness for the data sets is low, the data sets are >94% complete up to 1.3 and 1.6 Å, respectively. The less complete data in the higher-resolution shells still contribute to robust refinement and have been kept for this reason. The solvent-accessible surface area buried by the xylotriose portion of XUX was calculated in PyMOL by subtraction of area of the complex from the sum of the area of the protein and ligand separately (*i.e.* (*A*_protein_ + *A*_ligand_) − *A*_complex_).

### Intact protein MS

Observation of the covalent glucuronoyl-*Ot*CE15A H408A intermediate in solution was achieved by analysis of a cleavage reaction by MS using an Orbitrap Fusion Lumos mass spectrometer (Thermo Fisher Scientific) equipped with the heated electrospray ion source. Purified enzyme was dialyzed into 5 mm NH_4_HCO_3_, pH 7, prior to utilization in the reaction. A reaction mixture of 20 mg/ml *Ot*CE15A-H408A with 10 mm benzyl d-glucuronoate was mixed and left for 2 min before being quenched with 30% (v/v) methanol and 0.1% (v/v) formic acid. The quenched reaction mixture, containing ∼4 mg/ml protein, was analyzed via the direct infusion at 1 μl/min; alternatively, a protein sample without the added benzyl d-glucuronoate was analyzed to serve as the control. The samples were ionized in the positive mode at 3.0 kV, and analyzed in the Intact Protein mode using the low-resolution (15,000 target resolution) full MS scanning with five microscans in the precursor ranges 500–2000 or 1000–1200; alternatively, the high-resolution SIM scanning at 240,000 target resolution with 10 microscans and SIM isolation windows of 4, 6, and 12 was performed on the selected precursor ions. Higher-energy collision-induced dissociation spectra were recorded at 240,000 resolution and 25 normalized collision energy with 5 microscans in the range 100–2000 with the isolation window of 3.0 at the precursor ions with the *m*/*z* 1241, 1246, and 1251. Data were inspected using the Xcalibur Qual Browser viewer (Thermo Fisher Scientific); the high-resolution spectra were charge-deconvoluted using the Xtract feature in the Xcalibur to yield the monoisotopic masses of the proteoforms, and the low-resolution Orbitrap spectra were deconvoluted using the ESIprot online tool (45) to give the average molecular masses.

## Author contributions

S. M., L. L. L., and J. L. conceptualization; S. M., J.-C. N. P., L. L. L., and J. L. data curation; S. M., L. L. L., and J. L. validation; S. M., J.-C. N. P., L. L. L., and J. L. investigation; S. M. visualization; S. M., J.-C. N. P., L. L. L., and J. L. methodology; S. M., L. L. L., and J. L. writing-original draft; S. M., L. L. L., and J. L. project administration; S. M., L. L. L., and J. L. writing-review and editing; L. L. L. and J. L. resources; L. L. L. and J. L. supervision; L. L. L. and J. L. funding acquisition.

## Supplementary Material

Supporting Information

## Footnotes

4 The abbreviations used are:

GE: glucuronoyl esterase
CE15: carbohydrate esterase family 15
GlcA: glucuronate/glucuronic acid
GalA: galacturonate/galacturonic acid
BnzGlcA: benzyl glucuronoate
MeGlcA: methyl glucuronoate
MeGalA: methyl galacturonoate
XUX: 2^3^-(4-*O*-methyl-α-d-glucuronyl)-xylotriose
LCC: lignin-carbohydrate complex
PDB: Protein Data Bank.
