# Supplementary material for: Structural and biochemical studies of the glucuronoyl esterase OtCE15A illuminate its interaction with lignocellulosic components
Source: J Biol Chem. 2019 Nov 18;294(52):19978–87. doi: 10.1074/jbc.RA119.011435 (PMC6937553; doi:10.1074/jbc.RA119.011435)
Supplement: Supporting Information [file supp_294_52_19978__index.html]

Structural and biochemical studies of the glucuronoyl esterase OtCE15A illuminate its interaction with lignocellulosic components — Mechanism and ligand interactions of a glucuronoyl esterase — Structural and biochemical studies of the glucuronoyl esterase OtCE15A illuminate its interaction with lignocellulosic components — Mechanism and ligand interactions of a glucuronoyl esterase — Supporting Information 

# Structural and biochemical studies of the glucuronoyl esterase *Ot*CE15A illuminate its interaction with lignocellulosic components

## Supporting Information

- Supporting Information (to be published online) - Supporting information with kinetic data, primer sequences, crystallization and refinement data, multiple sequence alignment, and inhibition data.
