# Supplementary material for: Structural and biochemical studies of the glucuronoyl esterase OtCE15A illuminate its interaction with lignocellulosic components
Source: J Biol Chem. 2019 Nov 18;294(52):19978–87. doi: 10.1074/jbc.RA119.011435 (PMC6937553; doi:10.1074/jbc.RA119.011435)
Supplement: Supporting Information [file supp_RA119.011435_156397_2_supp_426884_q0r09l.pdf]

Table S1: Kinetic parameters of *Ot*CE15A on model substrates as previously reported (1). The model substrates utilized were benzyl D-glucuronoate (BnzGlcA), allyl D-glucuronoate (AllylGlcA), methyl D-glucuronoate (MeGlcA), methyl D-galacturonate (MeGalA), and *para*-nitrophenol acetate (*p*NP-Ac).

Substrate	K_m (mM)	k_{cat} (s^{-1})	k_{cat}/K_m ($s^{-1}M^{-1}$)
BnzGlcA	4.18 ± 0.14	19.4 ± 0.19	(4.64 ± 0.16) x 10^3
AllylGlcA	2.87 ± 0.10	25.2 ± 0.22	(8.80 ± 0.31) x 10^3
MeGlcA	2.77 ± 0.15	19.0 ± 0.31	(6.85 ± 0.39) x 10^3
MeGalA	5.31 ± 0.51	28.8 ± 0.64	(4.85 ± 0.47) x 10^3
*p*NP-Ac	*Cannot be saturated up to 10 mM*		(3.23 ± 0.063) x 10^1

Table S2. Primers utilized for creation of *Ot*CE15A variants and for cloning of the GH30 xylanase from *Bacteroides ovatus* (*Bo*GH30).

**Gene**	**Primer**	**5'-3' sequence**
*Ot*CE15A-S267A	S267A-f	GCATGCACGGCTCGGCAAG
	S267A-r	GCCGTGCATGCCCGTGC
*Ot*CE15A-H408A	H408A-f	CCGGCCGGAATGACCGCGC
	H408A-r	TCCGGCCGGCCCCGG
*Ot*CE15A-E290A	E290A-f	TCGAACGCATCCGGTTGCGG
	E290A-r	ACCGGATGCGTTCGAGATCACCAG
*Ot*CE15A-D356A	D356A-f	GAGGACGCTGATTGGGCGGATC
	D356A-r	CCAATCAGCGTCCTCGGCACTC
*Bo*GH30	*Bo*GH30-f	CTTCCAGGGCCATAGTTGTTCGGGAGGGGAAGATGAAAAAAAAG
	*Bo*GH30-r	TGGTGGTGCTCGAGTCTAAAATGTCAATCTGACTGAAGTTATACTGTTAGCA

Table S3. Crystallization conditions utilized for the respective datasets. Conditions were from, or optimized from, either Morpheus or JCSG+ screens (Molecular Dimensions) as described in the experimental procedures.

**PDB ID**	**Protein-ligand**	**Screen type**	**Condition**
6SYR	*Ot*CE15A-Wt-GlcA	Morpheus	0.1 M Carboxylic acids, 0.1 M Buffer System 3 pH 8.5, 37.5% v/v Precipitant Mix 4
6SZO	*Ot*CE15A-S267A-GalA	Morpheus	0.12 M Ethylene glycols, 0.1 M Buffer System 2 pH 7.5, 37.5% v/v Precipitant Mix 4
6SYV	*Ot*CE15A-S267A-GlcA	Morpheus	0.1 M Carboxylic acids, 0.1 M Buffer System 3 pH 8.5, 37.5% v/v Precipitant Mix 4
6T0I	*Ot*CE15A-Wt-XUX	Morpheus	0.06 M Divalents, 0.1 M Buffer System 1 pH 6.5, 37.5% v/v Precipitant Mix 4
6T0E	*Ot*CE15A-Wt-BnzGlcA	JCSG+	0.2 M TMAO, 0.1 M Tris pH 8.5, 20% w/v PEG 2000 MME
6SYU	*Ot*CE15A-Wt-Xylobiose	Morpheus	0.09 M NPS, 0.1 M Buffer System 3 pH 8.5, 37.5% v/v Precipitant Mix 4
6SZ0	*Ot*CE15A-H408A	Morpheus	0.09 M NPS, 0.1 M Buffer System 3 pH 8.5, 37.5% v/v Precipitant Mix 4
6SZ4	*Ot*CE15A-H408A-Acylated GlcA	JCSG+	0.2 M Ammonium formate, 20% w/v PEG 3350

Table S4a: Table of crystallographic statistics.

	*Ot*CE15A-Wt-GlcA	*Ot*CE15A-S267A-GlcA	*Ot*CE15A-S267A-BnzGlcA	*Ot*CE15A-S267A-GalA
Data Collection				
Date	December 9, 2018	October 6, 2018	December 09, 2018	September 18, 2018
Source	ID30A-3 at ESRF	ID23-2 at ESRF	ID30A-3 at ESRF	P11 at Petra III
Wavelength (Å)	0.9677	0.8731	0.9677	0.9891
Space group	P1	P1	$P2_12_12_1$	P1
Cell dimensions				
a, b, c (Å)	43.22, 44.19, 50.17	43.51, 44.50, 50.37	51.72, 87.61, 173.91	44.63, 46.14, 50.20
α, β, γ (°)	75.78, 65.42, 70.85	76.43, 66.96, 70.22	90, 90, 90	63.65, 86.79, 71.17
No. of measured reflections	177871 (18505)	334525 (15207)	865824 (79871)	44355 (4349)
No. of independent reflections	47249 (4751)	102117 (4777)	63514 (5944)	16711 (1620)
Resolution (Å)	32.39 - 1.49 (1.54 - 1.49)	33.85 - 1.12 (1.16 - 1.12)	44.45 - 1.89 (1.96 - 1.89)	44.74 - 2.20 (2.28 - 2.20)
R_{merge} (%)	6.13 (141.6)	8.79 (104.4)	15.9 (223.1)	8.13 (48.0)
$CC_{1/2}$ (%)	99.9 (65.6)	99.5 (45.2)	99.9 (49.9)	99.9 (92.5)
Mean I/σI	9.88 (0.82)	6.12 (0.76)	12.3 (0.84)	8.66 (2.00)
Completeness (%)	91.2 (91.4)	81.7 (38.3)	99.0 (92.0)	97.0 (94.6)
Redundancy	3.8 (3.9)	3.3 (3.2)	13.6 (13.4)	2.7 (2.7)
Refinement				
R_{work}/R_{free}	0.183/0.223	0.162/0.182	0.162/0.215	0.154/0.221
No. atoms				
Protein	3128	3247	6176	2782
Ligand/ions	31	99	344	194
Water	227	368	413	122
B-factors				
Protein	32.8	17.5	38.4	34.0
Ligand/ions	44.3	32.3	59.6	49.2
Water	39.7	28.9	43.2	38.6
RMSD				
Bond length (Å)	0.008	0.009	0.011	0.013
Bond angles (°)	1.05	1.18	1.15	1.29
PDB accession	6SYR	6SYV	6T0E	6SZO

Table S4b: Table of crystallographic statistics.

	*Ot*CE15A-Wt-XUX	*Ot*CE15A-Wt-Xylobiose	*Ot*CE15A-H408A	*Ot*CE15A-H408A-Acylated GlcA
Data Collection				
Date	December 9, 2018	November 8, 2018	May 29, 2019	May 29, 2019
Source	ID30A-3 at ESRF	BioMAX at MAXIV	P11 at Petra III	P11 at Petra III
Wavelength (Å)	0.9677	0.9184	1.0332	1.0332
Space group	P1	P1	P1	P1
Cell dimensions				
a, b, c (Å)	43.41, 44.17, 50.24	43.69, 44.34, 50.98	43.20, 44.20, 50.15	43.10, 44.15, 50.18
α, β, γ (°)	75.80, 65.49, 70.98	77.24, 67.27, 70.58	76.31, 65.70, 70.74	76.10, 66.23, 70.85
No. of measured reflections	158242 (12708)	203469 (3194)	105565 (8389)	104962 (9590)
No. of independent reflections	42565 (3634)	57996 (1682)	30758 (2499)	30662 (2844)
Resolution (Å)	32.49 - 1.54 (1.59 - 1.54)	41.58 - 1.33 (1.38 - 1.33)	45.39 - 1.74 (1.80 - 1.74)	45.56 - 1.75 (1.81 - 1.75)
R_{merge} (%)	4.43 (133)	4.83 (16.6)	4.63 (33.8)	9.83 (82.9)
$CC_{1/2}$	99.9 (50.8)	99.8 (94.2)	99.8 (94.5)	99.4 (73.6)
Mean I/σI	15.4 (0.72)	15.4 (3.40)	14.6 (2.85)	6.62 (1.53)
Completeness	88.6 (67.6)	77.0 (22.3)	94.2 (76.8)	95.5 (88.7)
Redundancy	3.7 (3.5)	3.5 (1.9)	3.4 (3.4)	3.4 (3.4)
Refinement				
$R_{\mathrm{work}}/R_{\mathrm{free}}$	0.160/0.195	0.120/0.145	0.142/0.192	0.214/ 0.284
No. atoms				
Protein	3109	3248	3138	3091
Ligand/ions	114	72	35	15
Water	258	626	223	109
B-factors				
Protein	29.2	12.4	31.4	38.4
Ligand/ions	44.6	27.7	49.3	36.1
Water	37.3	26.8	38.8	38.3
RMSD				
Bond length (Å)	0.010	0.008	0.009	0.012
Bond angles (°)	1.14	1.02	1.01	1.18
PDB accession	6T0I	6SYU	6SZ0	6SZ4

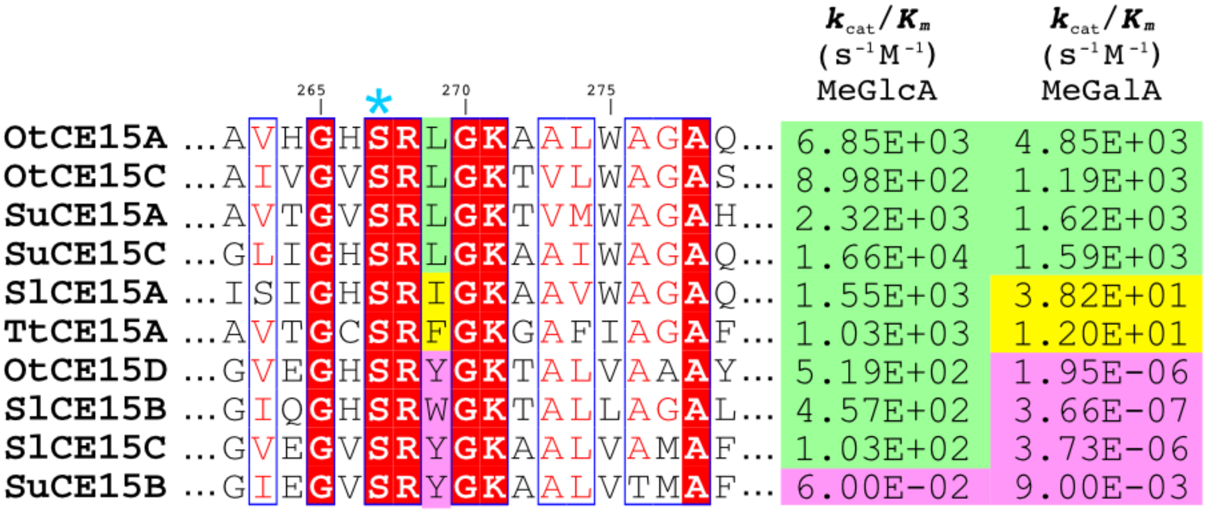

Figure S1. Correlation of a leucine residue and MeGalA activity in CE15 members. Excerpt of a multiple sequence alignment with select CE15 members from *Opitutus terrae* (Ot), *Solibacter usitatus* (Su), *Spirosoma linguale* (Sl), and *Teredinibacter turnerae* (Tt) shown together with their activities on MeGlcA and MeGalA. Amino acid numbering corresponds to the *Ot*CE15A enzyme, and the catalytic Ser residue is marked with a cyan asterisk. The leucine residue and high activity on a substrate is highlighted in green. Intermediately sized residues and reduced activity if highlighted yellow. Other residues found at the equivalent position and their associated diminished activity values are highlighted in magenta. The activity measurements are from previously determined results (1).

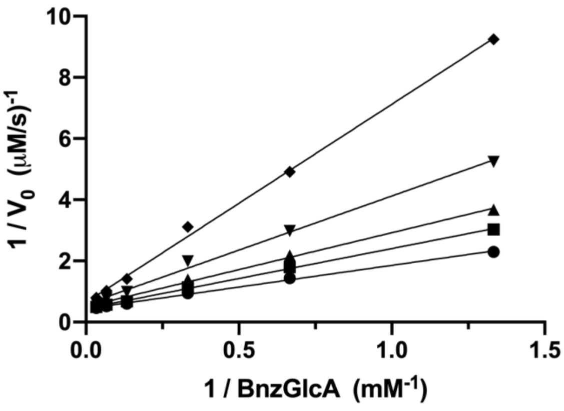

Figure S2. Lineweaver-Burk plot of xylose inhibition on the OtCE15A BnzGlcA esterase reaction. Kinetic assays were completed with xylose concentrations of either 0 mM (●), 30 mM (■), 60mM (▲), 125 mM (▼), or 250 mM (◆). The kinetic data was fitted in GraphPad Prism 8 to the competitive inhibition equation.

References

1. Arnling Bååth, J., Mazurkewich, S., Knudsen, R. M., Poulsen, J. N., Olsson, L., Lo Leggio, L., and Larsbrink, J. (2018) Biochemical and structural features of diverse bacterial glucuronoyl esterases facilitating recalcitrant biomass conversion. *Biotechnol Biofuels* **11**, 213
